# Impact of Genetic Polymorphisms of *SLC2A2*, *SLC2A5*, and *KHK* on Metabolic Phenotypes in Hypertensive Individuals

**DOI:** 10.1371/journal.pone.0052062

**Published:** 2013-01-14

**Authors:** MyPhuong T. Le, Maximilian T. Lobmeyer, Marcus Campbell, Jing Cheng, Zhiying Wang, Stephen T. Turner, Arlene B. Chapman, Eric Boerwinkle, John G. Gums, Yan Gong, Richard J. Johnson, Julie A. Johnson

**Affiliations:** 1 Department of Pharmacotherapy and Translational Research and Center for Pharmacogenomics, College of Pharmacy, University of Florida, Gainesville, Florida, United States of America; 2 Department of Epidemiology and Health Policy Research, College of Medicine, University of Florida, Gainesville, Florida, United States of America; 3 Human Genetics Center and Institute of Molecular Medicine, University of Texas Health Science Center, Houston, Texas, United States of America; 4 Division of Nephrology and Hypertension, Mayo Clinic, Rochester, Minnesota, United States of America; 5 Renal Division, Department of Medicine, Emory University, Atlanta, Georgia, United States of America; 6 Division of Renal Diseases and Hypertension, Department of Medicine, University of Colorado Denver, Aurora, Colorado, United States of America; The University of Texas Health Science Center, United States of America

## Abstract

**Objective:**

In the past few decades, consumption of added sugars has increased dramatically. Studies have linked high sugar intake with increased risk for a number of diseases. Importantly, fructose, a component of sugar, has been linked with the development of features of metabolic syndrome. This study determined if single nucleotide polymorphisms in genes involved in fructose transport (solute carrier family 2 facilitated glucose transporter, member 2 (*SLC2A2*) and solute carrier family 2 facilitated glucose/fructose transporter, member 5 (*SLC2A5*)) and metabolism (ketohexokinase (*KHK*)) affect inter-individual variability in metabolic phenotypes, such as increased serum uric acid levels.

**Materials/Methods:**

The influence of *SLC2A2*, *SLC2A5*, and *KHK* SNPs on metabolic phenotypes was tested in 237 European Americans and 167 African Americans from the Pharmacogenomic Evaluation and Antihypertensive Responses (PEAR) study. Using baseline untreated fasting data, associations were considered significant if p≤0.005. These SNPs were then evaluated for potential replication (p≤0.05) using data from the Genetic Epidemiology of Responses to Antihypertensives (GERA) studies.

**Results:**

*SLC2A5* rs5438 was associated with an increase in serum uric acid in European American males. However, we were unable to replicate the association in GERA. The minor allele of *SLC2A2* rs8192675 showed an association with lower high-density lipoproteins in European Americans (A/A: 51.0 mg/dL, A/G: 47.0 mg/dL, G/G: 41.5 mg/dL, p = 0.0034) in PEAR. The association between rs8192675 and lower high-density lipoproteins was replicated in the combined European American GERA study samples (A/A: 47.6 mg/dL, A/G: 48.6 mg/dL, G/G: 41.9 mg/dL, p = 0.0315).

**Conclusions:**

The association between *SLC2A2* rs8192675 and high-density lipoproteins suggests the polymorphism may play a role in influencing high-density lipoproteins and thus metabolic risk of cardiovascular disease.

## Introduction

There has been a remarkable increase in the intake of added sugars. In the 1970s, an individual's sugar intake in the United States was approximately 120 lbs per year. This increased approximately to 150 lbs per year in 2000 [1], [2], [3]. Recent studies have associated higher added sugar consumption, especially from sugar-sweetened beverages, with increased risk for the development of obesity, hyperuricemia, hypertension, metabolic syndrome, type 2 diabetes, and cardiovascular diseases [4]–[11]. Of particular interest is the growing number of studies implicating fructose, a component of added sugars such as sucrose and high fructose corn syrup, as a factor in the pathogenesis and prevalence of these diseases.

Experimental studies have shown that fructose consumption can increase adipogenesis, stimulate lipogenesis, increase triglycerides and apoB levels, increase blood pressure, decrease insulin sensitivity, and suppress leptin levels [12], [13], [14], [15], [16]. Compared to other natural sugars, fructose also has the unique ability to increase uric acid levels, which has been proposed to have a contributory role in hypertension and cardiovascular disease [17], [18]. Because fructose has been shown to induce a variety of adverse metabolic effects, this has led some to suggest that fructose may play an important role in driving obesity and cardiovascular diseases [19], [20].

Based on the ability of fructose to induce features of the metabolic syndrome, we hypothesized that polymorphisms in genes involved in fructose transport and metabolism might influence metabolic phenotypes in subjects with established hypertension. Two fructose transporters were chosen: glucose transporter type 5 (GLUT5) and glucose transporter type 2 (GLUT2), which are encoded by *SLC2A5* and *SLC2A2*, respectively. GLUT5 is the primary transporter facilitating the diffusion of fructose across the apical membrane of enterocytes after its ingestion [21]. Although GLUT2 prefers to transport glucose, recent studies have shown that the protein also has an important role in the absorption and transport of fructose [22], [23]. We also evaluated the enzyme ketohexokinase, which mediates the initial metabolism of fructose and is encoded by the gene *KHK* [24]. Because of their role in fructose homeostasis, we hypothesized single nucleotide polymorphisms (SNPs) in these three candidate genes may potentially influence an individual's response to fructose; and thus, impart variability in individual risk for developing adverse metabolic effects and the development of diseases.

## Methods

### Ethic Statement

While PEAR's protocol allowed the recruitment of minors, the youngest participant was 21 years old. The study protocol for PEAR was approved by the institutional review boards of University of Florida, Emory University, and Mayo Clinic. The study protocols for the GERA studies were approved by the institutional review boards of Emory University and Mayo Clinic. All subject provided informed written consent.

### Study Populations

Data collected from the Pharmacogenomic Evaluation and Antihypertensive Responses study (PEAR) and the Genetic Epidemiology of Responses to Antihypertensives studies (GERA I and II) were utilized [25], [26], [27]. The objectives of all three studies were to investigate whether genetic polymorphisms predicted the inter-individual variability in blood pressure and adverse metabolic responses to antihypertensive drugs. PEAR recruited mild to moderate essential hypertensives, male or female, of any race or ethnicity, and between the ages of 17 and 65 [25]. The GERA studies recruited males and females with essential hypertension between the ages of 30 and 59 [26], [27]. The three studies excluded subjects who had diabetes mellitus, liver failure, or kidney disease. Atenolol and hydrochlorothiazide were used to treat PEAR subjects. Hydrochlorothiazide was also used for GERA I. For GERA II, candesartan was the antihypertensive drug. All three studies had a wash-out period of approximately one month for patients to reach a baseline before the initiation of antihypertensive treatments.

For this exploratory association study, we only utilized baseline untreated fasting laboratory data collected from the three study populations. PEAR was used as the discovery cohort while GERA I and II were used as the replication cohorts for associations for the three candidate genes. This analysis was conducted when PEAR was ongoing and a total of 418 subjects were genotyped for this project. Only data from African Americans and European Americans were used for the association analyses, thus eliminating 14 subjects belonging to other racial/ethnic groups. GERA I recruited 289 African Americans and 295 European Americans. GERA II recruited 252 African Americans and 300 European Americans. We utilized data from existing genome-wide association studies in GERA, where only good and poor responders in the GERA studies were genotyped [28]. For GERA I, 194 African Americans and 196 European Americans were genotyped. For GERA II, 193 African Americans and 198 European Americans were genotyped.

### Genotyping

PEAR was genotyped using the HumanCVD Beadchip (Illumina Inc., San Diego, CA) [25], [29]. There were 13 *SLC2A2* SNPs, 11 *SLC2A5* SNPs, and 5 SNPs of *KHK* on the chip. In order to increase the characterization of the three candidate genes, MultiPop-TagSelect algorithm (minor allele frequency (MAF) cutoff = 5%, r^2^ = 0.8), cosmopolitan tagSNPs were selected to best capture the genetic variability in *SLC2A2*, *SLC2A5*, and *KHK* in HAPMAP's European (CEU) and African (YRI) populations [30]. In addition, *in-silico* analyses were used to assess the SNPs reported in the National Center for Biotechnology Information's dbSNP database (Build 128) [31], [32]. SNPs determined to be putatively functional (pfSNP) by PupaSuite and FASTSNP were also included [33], [34]. A custom genotyping array (oligo pool all, OPA) was successfully designed containing 31 SNPs: 11 *SLC2A2* SNPs, 19 *SLC2A5* SNPs, and 1 *KHK* SNP. Table S1 lists all the SNPs that were genotyped in PEAR.

Illumina's Infinium® II, a whole genome bead-chip genotyping technology, was used to assay the HumanCVD chips [35]. The PEAR DNA samples were processed according to the manufacturer's protocol. The intensities of the fluorescence were detected by the Illumina BeadArray Reader. The OPA chip was assayed using Illumina's Veracode™ GoldenGate® chemistry and the bead plates were scanned by Ilumina's BeadXpress Station. The image files from the HumanCVD and OPA chips were then analyzed by Illumina's BeadStudio Genotyping Analysis Module 3.3.7.

Genotyping for GERA I and II had already been completed. GERA I was genotyped using the GeneChip Human Mapping 100K array set and GERA II was genotyped with the genome-wide human SNP array 6.0 (Affymetrix, Santa Clara, CA) [28], [36].

### Data Analysis

#### Quality control procedures

Samples were excluded if they were contaminated, had sex-gender-estimate mismatches, or had low call rates (<95% for HumanCVD chip, <90% for OPA chip). SNPs, which were clustered using a Gen Call Threshold of 0.15 for the HumanCVD chip and 0.25 for the OPA chip, were removed if they had poor clustering scores (GenTrainScore <0.3), had call rates <95%, or were monomorphic. From the Hardy-Weinberg Equilibrium (HWE) analysis, SNPs were excluded if the chi-square test p-values were <0.05 in both African American and European American populations. In addition, SNPs with a MAF<5% in both races were excluded from analysis due to lack of power. For SNPs with a MAF of 5%, there was a greater than 91% power to detect an effect size of 1.0; and for MAF of 20%, there was a greater than 99% power to detect effect size of 0.8 (α = 0.005, n = 200 by race) [37], [38]. 48 SNPs were analyzed (18 *SLC2A2*, 28 *SLC2A5*, and 2 *KHK*).

#### SNP-trait associations

Due to the strong experimental link between fructose and uric acid, the primary adverse phenotype was fasting serum uric acid (SUA) [39], [40]. Secondary phenotypes were triglycerides (TG), and high-density lipoprotein (HDL) [12], [13], [14]. Because of the differences in linkage disequilibrium structures and frequencies of variant alleles, the data were stratified for analysis by race. The interaction between sex and SNP were also analyzed for SUA and HDL levels because of sex differences in these metabolic parameters [41], [42], [43]. If the interaction was significant, the data were also stratified by sex. Furthermore, PEAR subjects who were assessed to be not fasting at the study visits based on glucose, insulin and triglycerides at several visits were excluded.

For each adverse metabolic phenotype, analysis of variance (ANOVA) was performed while controlling for age, BMI, and/or sex. Assuming 144 independent tests were conducted, a Bonferroni correction for multiple comparisons would require a p≤0.00035 for significance (0.05/144 = 0.00035). Due to linkage disequilibrium between the SNPs of the candidate genes, the assumption of independence between comparisons for a Bonferroni correction was considered too conservative. Therefore to avoid missing potential true associations as well as avoid keeping possibly false associations, we considered two sequential association studies to identify SNPs associated with SUA, TG, or HDL. SNPs with an adjusted p-value≤0.005 were considered significant in the initial discovery process in PEAR. To confirm these findings, a replication study was conducted in GERA I and II and a SNP-trait association was considered significant if the p-value was ≤0.05. Thus, with matching directions of effect, the overall p-value for significance was 0.005×0.05×0.5 = 0.000125, which therefore exceeded the Bonferroni threshold for significance [44].

SNP-trait association analyses were performed for each gene using SAS 9.2 and JMP Genomics 4.0 (SAS Institute Inc., Cary, NC). For GERA I and II, the analyses were conducted by the studies' central data coordinating center.

## Results

### Baseline Demographics and Data Quality Control

Baseline characteristics for PEAR and GERA study samples are shown in Table 1. The subjects in the three study samples had similar characteristics, such as glucose, DBP, SBP, and BMI. However, TG showed the greatest variability. After quality control, 10 SNPs were excluded: 2 SNPs with call rates <95%, 4 SNPs with MAF<5% in both African and European Americans, and 4 SNPS that were monomorphic. 25 PEAR samples were excluded: 14 non-European or African Americans, 1 sample with low DNA yield and quality, 4 samples with a low call rate, 1 sample with sex-gender mismatch, and 11 (8 African Americans and 3 European Americans) nonfasting samples. This left 48 SNPs to be tested in 393 African and European Americans.

**Table 1:** Baseline characteristics of study populations.

	PEAR	GERA I	GERA II
	(n = 418)	(n = 390 )	(n = 391)
Age	49.5±8.8	47.9±6.8	48.9±6.7
Female	236 (56.5)	195 (50)	195 (49.9)
Race			
White, European American	237 (56.7)	196 (50.3)	198 (50.6)
Black, African American	167 (40.0)	194 (49.7)	193 (49.4)
Asian	5 (1.2)		
Other/Multiracial	9 (2.1)		
Primary Phenotypes			
SUA (mg/dL)	5.6±1.4	5.5±1.4	no data
Secondary Phenotypes			
DBP (mmHg)	98.6±6.7	96.4±5.6	95.1±5.2
SBP (mmHg)	152.2±13.0	146.7±14.4	147.2±12.5
Glucose (mg/dL)	92.3±11.6	94.8±13.7	97.0±13.8
HDL (mg/dL)	49.3±14.2	46.0±13.8	51.8±15.1
LDL (mg/dL)	122.7±30.3	107.6±31.9	115.6±32.6
TG (mg/dL)	127.7±95.4	153.7±86.0	134.3±110.8
BMI (kg/m^2^)	31.0±5.7	31.4±6.2	30.1±4.3

BMI body mass index; DBP diastolic blood pressure (office); HDL high-density lipoprotein; SBP systolic blood pressure (office); LDL low-density lipoprotein; TG triglycerides; SUA serum uric acid. GERA Genetic Epidemiology of Responses to Antihypertensives; PEAR Pharmacogenomic Evaluation and Antihypertensive Responses. Data are given as mean ± standard deviation or n (%).

### Primary Phenotypes

#### SUA

**Figure 1:**
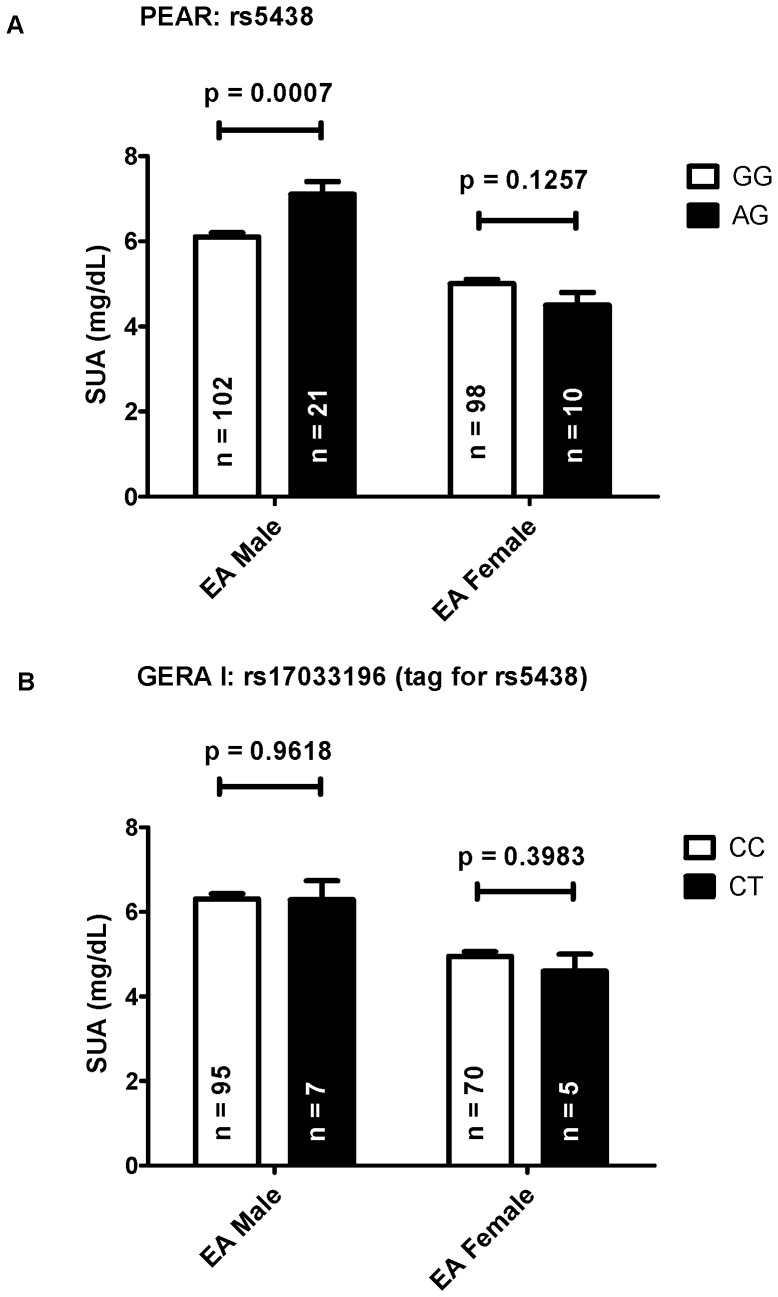
Effects of rs5438 and rs17033196 on SUA levels. A. Effects of rs5438 on SUA levels in European Americans (EA) of PEAR. B. Effects of rs17033196 (tagged rs5438 r2 = 1) on SUA levels in European Americans of GERA I. ANOVA was adjusted for age and BMI and shown as least square mean ± standard error.

One SNP, *SLC2A5* rs5438, was associated with SUA, and exhibited a significant SNP x sex interaction (p = 0.0011) leading to analyses stratified by sex. Rs5438 was found to be significantly associated with baseline SUA in only European American males (Table 2, Figure 1A). 21 or 17.1% of white men carried one copy of the minor allele, which was associated with approximately 1.1 mg/dL higher serum uric acid concentrations.

**Table 2:** SNPs significantly associated with baseline traits in PEAR.

					Genotype						
					HCA		HET		HMA		
Gene	Marker	Variant	Trait	Race	n (%)	Mean (mg/dL)	n (%)	Mean (mg/dL)	n (%)	Mean (mg/dL)	P-value
*SLC2A5*	rs5438	G>A	SUA	EA:	102 (82.9)	6.1±1.2	21 (17.1)	7.2±1.4			0.0007^B^
				Male		6.1±0.1		7.1±0.3			
*SLC2A2*	rs11924032	G>A	TG	EA	124 (53.2)	131.2±69.1	97 (41.6)	158.0±109.9	12 (5.2)	209.2±190.5	0.0038^A^
						128.6±8.5		157.8±9.6		211.3±27.1	
*SLC2A2*	rs5398	C>T	TG	EA	111 (48.3)	128.6±69.6	104 (45.2)	160.5±107.3	15 (6.5)	197.8±172.2	0.0046^A^
						126.8±9.0		159.4±9.3		197.0±24.3	
*SLC2A2*	rs8192675	A>G	TG	EA	114 (48.9)	127.6±69.0	104 (44.6)	160.5±107.3	15 (6.4)	197.8±172.2	0.0034^A^
						126.0±8.8		159.4±9.3		197.1±24.2	
*SLC2A5*	rs12086036	A>G	TG	AF	75 (51.0)	88.7±46.7	62 (42.2)	94.2±69	10 (6.8)	202.3±249.5	0.0043^A^
						97.4±10.4		104.1±11.5		194.5±26.5	
*SLC2A2*	rs5398	C>T	HDL	EA	111 (48.3)	50.6±14.2	104 (45.2)	46.8±13.3	15 (6.5)	41.5±12.2	0.0028^A^
						51.2±1.2		47.0±1.2		41.5±3.1	
*SLC2A2*	rs8192675	A>G	HDL	EA	114 (48.9)	50.4±14.1	104 (44.6)	46.8±13.3	15 (6.4)	41.5±12.2	0.0034^A^
						51.0±1.1		47.0±1.2		41.5±3.1	

A adjusted for age, BMI, and sex;

B adjusted for age and BMI; PEAR Pharmacogenomic Evaluation and Antihypertensive Responses.

AF African American; EA European American. HDL high-density lipoprotein; SUA serum uric acid; TG triglycerides. HCA homozygote common allele; HET heterozygote; HMA homozygote minor allele. Analysis of covariance conducted for SNP-trait associations. P-value≤0.005 was considered significant. Adjusted data are given as least square mean ± standard error.

Rs17033196, which tagged rs5438 in European Americans with an r^2^ = 1, was imputed from HapMap 2 data with an imputation score of r^2^ = 0.72 [45]. In GERA I study population, there were no significant interaction between this SNP and sex (p = 0.6249) or association with SUA levels (Figure 1B). No uric acid data were available in GERA II.

### Secondary Phenotypes

#### HDL

**Figure 2:**
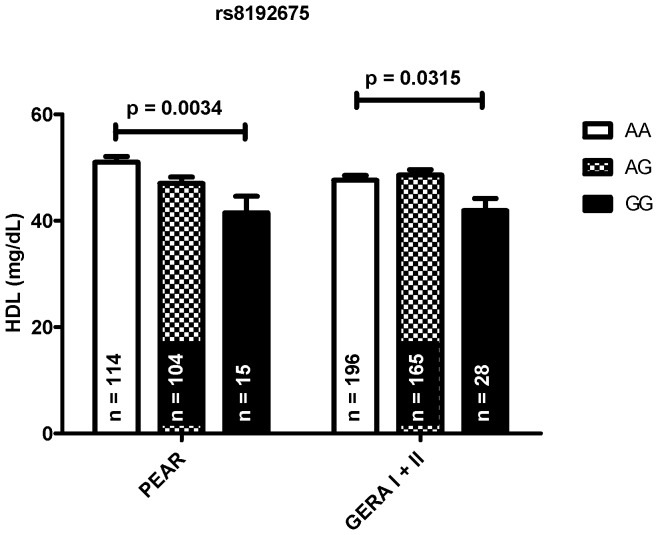
Effects of rs8192675 on HDL levels in PEAR and GERA European Americans. ANOVA was adjusted for age, BMI, and sex and shown as least square mean ± standard error.

After adjusting for covariates, two *SLC2A2* SNPs were associated with HDL in European Americans (Table 2). Individuals that were carriers of one copy of the minor allele of either rs5398 or rs8192675 showed a decrease in HDL levels by about 4 mg/dL. As a tagSNP for rs5398 (r^2^ = 1) in European Americans, rs8192675 was significantly associated with HDL in the combined GERA study populations (Figure 2). In GERA, minor allele homozygotes exhibited an approximately 6 mg/dL decrease in HDL (A/A: 47.6 mg/dL, A/G: 48.6 mg/dL, G/G: 41.9 mg/dL).

#### TG

After adjusting for age, BMI, and sex, four SNPs (*SLC2A2*: rs11924032, rs5398, and rs8192675; *SLC2A5*: rs12086036) were significantly associated with TG with p-values≤0.005 (Table 2). The associations for all three *SLC2A2* SNPs were observed only in European Americans. All three minor alleles appeared to have an additive effect on TG levels. With each copy, TG increased by about 30 mg/dL. None of these SNP associations for TG replicated in GERA I and/or GERA II.

For *SLC2A5* rs12086036, the SNP's association was in African Americans only. Two copies of the minor allele (GG) appeared to increase TG levels by about 95 mg/dL. There were no genotyping data available for this SNP in GERA, so further replication was not possible.

## Discussion

From the discovery cohorts, several potentially interesting associations were detected between genetic polymorphisms of *SLC2A2* and *SLC2A5* with various metabolic phenotypes, including serum uric acid, TG, and HDL. Importantly, *SLC2A2* rs8192675, which tagged rs5398, was associated with both TG and HDL levels in the European American subjects in PEAR and was also significantly associated with a lower HDL levels in the combined European American GERA cohorts. This represents the most compelling finding of the study and suggests the potential role of the glucose and fructose transporter GLUT2 on HDL levels.

Associated with HDL and TG levels in PEAR, *SLC2A2* rs8192675 is located in intron 5. Rs5398, tagged by rs8192675, causes a synonymous polymorphism and is located in exon 11. Although the functional consequences of these polymorphisms are unknown, mutations of *SLC2A2*, which is highly expressed in the liver and the β-cell islets, cause Fanconi-Bickel syndrome. This disease is associated with hyperglycemia, hypoinsulinemia, and hypertriglyceridemia [46], [47], [48], [49], [50]. Polymorphisms of *SLC2A2* have also been linked with increased risk of developing type 2 diabetes [46], [50]. Rs5393 and rs5400 were pfSNPs in this study, but both failed to be significantly associated with glucose levels, which may have been impacted by the exclusion of diabetics from both PEAR and GERA. Importantly, recent studies have implicated insulin-resistance and hypertriglyceridemia with the lowering of HDL levels, potentially through the increased metabolism of apoA-I, an essential component of HDL particles [49], . Thus, while the potential functional mechanism of our findings is not clear, the literature suggests such phenotypic associations could be consistent with altered function of the GLUT2 transporter. Because GLUT2 can transport glucose and fructose, polymorphisms in *SLC2A2* can affect the homeostasis of both carbohydrates; and thus, may magnify the development of adverse phenotypes. Further studies are needed to elucidate if these polymorphisms influence HDL by altering fructose and/or glucose levels.

We were also able to discover associations between SNPs of *SLC2A2* and *SLC2A5* with serum uric acid and/or triglycerides in PEAR. However, we were unable to replicate our findings in GERA. Our inability to replicate our findings may be due to several reasons. For serum uric acid, the frequency of heterozygotes in the PEAR population was about 17% while the GERA I population only had a frequency of 7%. This discrepancy may be due to the use of imputed genotype data in GERA, where the imputation score for rs17033196 was not optimal (0.72). As for triglycerides, fasting levels have been reported to be highly variable [54]. Unlike the other baseline characteristics, fasting triglycerides differed up to about 20% between the three study populations. Furthermore, recent genome-wide association studies have detected significant findings with serum uric acid levels, lipids, and BMI [55], [56]. However, the effect sizes detected have been small; thus, highlighting the potential lack of power in our study due to the small sample size

In conclusion, this exploratory study detected some potentially interesting associations between polymorphisms of *SLC2A2* and *SLC2A5* with various aspects of the metabolic syndrome in the study population of PEAR. The data suggest that these genes, especially *SLC2A2*, may have an important role in increasing an individual's risk for developing adverse metabolic phenotypes, such as decreased HDL levels. More intensive studies are needed in larger populations to better characterize the impact of polymorphisms of *SLC2A2*, *SLC2A5*, and *KHK* on the development of adverse metabolic effects and increased disease risks, especially since recent studies have linked SNPs of *SLC2A2* to the development of type 2 diabetes. Along with better coverage of these genes, the interesting SNPs from this study can be utilized as candidate SNPS in future studies.

## Supporting Information

Table S1 **Genotype results of PEAR samples using HumanCVD and OPA genotyping chips.**
(DOCX)
