# Supplementary material for: Impact of Genetic Polymorphisms of SLC2A2, SLC2A5, and KHK on Metabolic Phenotypes in Hypertensive Individuals
Source: PLoS One. 2013 Jan 14;8(1):e52062. doi: 10.1371/journal.pone.0052062 (PMC3544854; doi:10.1371/journal.pone.0052062)
Supplement: Table S1 — Genotype results of PEAR samples using HumanCVD and OPA genotyping chips. (DOCX) [file pone.0052062.s001.docx]

Table S1. Genotype results of PEAR samples using HumanCVD and OPA genotyping chips.

| **Gene** | **rs#** | **CP** |  | **Variant** |  | **African Americans** | |  | **European Americans** | |
| --- | --- | --- | --- | --- | --- | --- | --- | --- | --- | --- |
|  |  |  | **Genotype Chip** |  |  | **MA** | **MAF** |  | **MA** | **MAF** |
| *SLC2A2* | rs10513684 | 172206904 | CVD | C/T |  | T | 0.03 |  | T | 0.05 |
|  | rs10513685 | 172207084 | CVD | A/G |  | A | 0.39 |  | A | 0.13 |
|  | rs10513688^E^ | 172209912 | OPA | A/G |  | A | 0.3 |  | A | 0.13 |
|  | rs11711206 | 172215639 | OPA | A/G |  | G | 0.29 |  | G | 0.11 |
|  | rs11711437 | 172209057 | CVD | C/G |  | C | 0.48 |  | G | 0.14 |
|  | rs11924032 | 172217793 | CVD | A/G |  | A | 0.4 |  | A | 0.26 |
|  | rs11924648^A^ | 172200690 | OPA | A/G |  | G | 0.43 |  | G | 0.14 |
|  | rs11925298^P^ | 172219412 | CVD, OPA | A/G |  | A | 0.11 |  | A | 0.02 |
|  | rs12487486 | 172200825 | OPA | C/T |  | T | 0.39 |  | T | 0.14 |
|  | rs12488694 | 172227055 | CVD | C/T |  | T | 0.05 |  | T | 0.02 |
|  | rs16855638^A^ | 172201058 | CVD | G/T |  | G | 0.1 |  | G | 0 |
|  | rs3774046 | 172219697 | OPA | C/T |  | C | 0.09 |  | C | 0.16 |
|  | rs5393^P^ | 172227614 | OPA | A/C |  | C | 0.49 |  | C | 0.13 |
|  | rs5396^P^ | 172227509 | CVD | A/G |  | A | 0.34 |  | G | 0.29 |
|  | rs5398 | 172198524 | OPA | C/T |  | C | 0.39 |  | T | 0.29 |
|  | rs5400^A,^ ^P^ | 172214994 | CVD | C/T |  | T | 0.46 |  | T | 0.14 |
|  | rs8192675 | 172207577 | OPA | A/G |  | A | 0.27 |  | G | 0.29 |
|  | rs9828378 | 172226766 | CVD | C/G |  | G | 0.11 |  | G | 0.15 |

CP chromosomal position; CVD HumanCVD genotyping chip; HWE Hardy-Weinberg Equilibrium; MA minor allele; MAF minor allele frequency; OPA oligo pool all custom SNP genotyping chip. ^P^ putatively functional SNP. Hardy-Weinberg Equilibrium: ^A^ p-value <0.05 in African Americans; ^E^ p-value < 0.05 in European Americans.

Table S1. Continued.

| **Gene** | **rs#** | **CP** | **Genotype Chip** | **Variant** |  | **African Americans** | |  | **European Americans** | |
| --- | --- | --- | --- | --- | --- | --- | --- | --- | --- | --- |
|  |  |  |  |  |  | **MA** | **MAF** |  | **MA** | **MAF** |
| *SLC2A5* | rs1060998 | 9023066 | OPA | C/T |  | T | 0.16 |  | T | 0.27 |
|  | rs1063137^P^ | 9019838 | OPA | C/T |  | C | 0.47 |  | T | 0.36 |
|  | rs12025713 | 9017107 | OPA | C/T |  | C | 0.49 |  | C | 0.36 |
|  | rs12068539^P^ | 9017816 | OPA | A/G |  | A | 0.49 |  | A | 0.36 |
|  | rs12080175 | 9039330 | OPA | C/T |  | C | 0.12 |  | C | 0.27 |
|  | rs12086036^P^ | 9043612 | OPA | A/G |  | G | 0.28 |  | G | 0.28 |
|  | rs12117043 | 9055107 | OPA | A/G |  | A | 0.15 |  | A | 0.32 |
|  | rs12119987 | 9016477 | OPA | A/T |  | A | 0.49 |  | A | 0.35 |
|  | rs12145292 | 9041183 | CVD | A/C |  | A | 0.29 |  | A | 0.26 |
|  | rs12736085 | 9053237 | OPA | A/G |  | G | 0.32 |  | G | 0.27 |
|  | rs1612895 | 9045144 | CVD | A/G |  | A | 0.29 |  | G | 0.44 |
|  | rs1705295 | 9044969 | CVD | C/T |  | T | 0.01 |  | T | 0.11 |
|  | rs1751680^P^ | 9044989 | OPA | C/T |  | T | 0.26 |  | T | 0.37 |
|  | rs17842190 | 9041010 | OPA | C/T |  | T | 0.05 |  | T | 0.02 |
|  | rs2478868^P^ | 9049803 | OPA | G/T |  | C | 0.31 |  | C | 0.36 |
|  | rs2505972 | 9039486 | CVD | A/C |  | A | 0.3 |  | C | 0.38 |
|  | rs3004245 | 9034213 | OPA | A/C |  | A | 0.26 |  | C | 0.45 |
|  | rs3737661 | 9030436 | CVD | A/C |  | A | 0.1 |  | A | 0.07 |
|  | rs3765962 | 9050045 | CVD | A/T |  | T | 0.14 |  | T | 0.29 |
|  | rs3820034^P^ | 9052454 | OPA | C/T |  | T | 0.03 |  | T | 0.2 |
|  | rs4908526 | 9025544 | OPA | C/T |  | T | 0.48 |  | T | 0.4 |
|  | rs4908809 | 9038287 | OPA | C/T |  | C | 0.43 |  | C | 0.36 |
|  | rs5438^P^ | 9052207 | OPA | A/G |  | A | 0.13 |  | A | 0.07 |
|  | rs6680123 | 9040335 | CVD | C/T |  | C | 0.11 |  | C | 0.01 |
|  | rs6694527 | 9041615 | CVD | A/G |  | A | 0.09 |  | G | 1 |
|  | rs7521322 | 9036678 | CVD | A/G |  | G | 0.49 |  | G | 0.39 |
|  | rs765617 | 9044369 | CVD | C/T |  | T | 0.44 |  | T | 0.33 |
|  | rs770041^P^ | 9052532 | CVD | A/G |  | A | 0.16 |  | A | 0.34 |

CP chromosomal position; CVD HumanCVD genotyping chip; HWE Hardy-Weinberg Equilibrium; MA minor allele; MAF minor allele frequency; OPA oligo pool all custom SNP genotyping chip. ^P^ putatively functional SNP. Hardy-Weinberg Equilibrium: ^A^ p-value <0.05 in African Americans; ^E^ p-value < 0.05 in European Americans.

Table S1. Continued.

| **Gene** | | **rs#** | | | **CP** | | | | **Genotype Chip** | | | | | | | **Variant** | | | | **African Americans** | | | | | | | |  | | **European Americans** | | | |  |
| --- | --- | --- | --- | --- | --- | --- | --- | --- | --- | --- | --- | --- | --- | --- | --- | --- | --- | --- | --- | --- | --- | --- | --- | --- | --- | --- | --- | --- | --- | --- | --- | --- | --- | --- |
|  |  |  |  |  |  |  |  |  |  |  |  |  |  |  |  |  |  |  |  |  | **MA** | | **MAF** | | | |  | | **MA** | | | **MAF** | | |
| *KHK* | | rs1131375 | | | 27176889 | | | | CVD | | | | | | | C/T | | | |  | T | | 0.15 | | | |  | | T | | | 0.36 | | |
|  | | rs7588333 | | | 27165775 | | | | CVD | | | | | | | C/G | | | |  | C | | 0.09 | | | |  | | C | | | 0 | | |
|  | |  | | |  | | | | |  | | | | | |  | | | |  |  | | |  | |  | | | |  | |  |  |  |
|  | |  | | |  | | | |  | | | | | | | **EXCLUDED SNPS** | | | |  |  | |  | | | |  | |  | | |  | | |
| **Call Rate < 95%** | | | |  | | |  |  | |  | | |  | |  | | |  | |  | |  | | | |  |  |  |  |  |  |  |  |  |
| *KHK* | | rs2304681^P^ | | | 27168756 | | | | CVD, OPA | | | | | | | A/G | | | |  | A | | 0.31 | | | |  | | A | | | 0.36 | | |
| *SLC2A5* | | rs3004249^E^ | | | 9043984 | | | | OPA | | | | | | | A/G | | | |  | A | | 0.45 | | | |  | | G | | | 0.39 | | |
|  | |  | | |  | | | | |  | | | | | |  | | | |  |  | | |  | |  | | | |  | |  |  |  |
| **MAF < 5%** | | | |  | | |  |  | |  | | |  | |  | | |  | |  | |  | | | |  |  |  |  |  |  |  |  |  |
| *KHK* | | rs36045790 | | | 27161663 | | | | CVD | | | | | | | C/T | | | |  | C | | 1 | | | |  | | T | | | 0.03 | | |
| *KHK* | | rs7561117 | | | 27165936 | | | | CVD | | | | | | | A/G | | | |  | A | | 0.04 | | | |  | | G | | | 1 | | |
| *SLC2A2* | | rs5397 | | | 172198529 | | | | CVD | | | | | | | C/G | | | |  | C | | 1 | | | |  | | G | | | 0 | | |
| *SLC2A2* | | rs7610064 | | | 172198030 | | | | CVD | | | | | | | A/T | | | |  | T | | 1 | | | |  | | A | | | 0.01 | | |
|  | |  | | |  | | | |  | | | | | | |  | | | |  |  | |  | | | |  | |  | | |  | | |
| **Monomorphic SNPS** | | | | | | |  | | | |  |  | |  | |  | | |  | | | |  |  |  |  |  |  |  |  |  |  |  |  |
| *SLC2A2* | | rs2229608 | | | 172198839 | | | | CVD | | | | | | | C/T | | | |  | T | | 1 | | | |  | | T | | | 1 | | |
| *SLC2A2* | | rs5395^P^ | | | 172227514 | | | | OPA | | | | | | | C/T | | | |  | T | | 1 | | | |  | | T | | | 1 | | |
| *SLC2A2* | | rs5408^P^ | | | 172199641 | | | | OPA | | | | | | | C/T | | | |  | T | | 1 | | | |  | | T | | | 1 | | |
| *SLC2A5* | | CT_9011003 | | | 9011003 | | | | OPA | | | | | | | C/T | | | |  | T | | 1 | | | |  | | T | | | 1 | | |

CP chromosomal position; CVD HumanCVD genotyping chip; HWE Hardy-Weinberg Equilibrium; MA minor allele; MAF minor allele frequency; OPA oligo pool all custom SNP genotyping chip. ^P^ putatively functional SNP. Hardy-Weinberg Equilibrium: ^A^ p-value <0.05 in African Americans; ^E^ p-value < 0.05 in European Americans.
